# The autoinflammation-associated NLRC4^V341A^ mutation increases microbiota-independent IL-18 production but does not recapitulate human autoinflammatory symptoms in mice

**DOI:** 10.3389/fimmu.2023.1272639

**Published:** 2023-11-28

**Authors:** Elien Eeckhout, Tomoko Asaoka, Hanne Van Gorp, Dieter Demon, Charlotte Girard-Guyonvarc’h, Vanessa Andries, Lars Vereecke, Cem Gabay, Mohamed Lamkanfi, Geert van Loo, Andy Wullaert

**Affiliations:** ^1^ Department of Internal Medicine and Pediatrics, Ghent University, Ghent, Belgium; ^2^ VIB-UGent Center for Inflammation Research, VIB, Ghent, Belgium; ^3^ Division of Rheumatology, Department of Medicine, University Hospital of Geneva, Department of Pathology and Immunology, University of Geneva Faculty of Medicine, Geneva, Switzerland; ^4^ Department of Biomedical Molecular Biology, Ghent University, Ghent, Belgium; ^5^ Laboratory of Proteinscience, Proteomics and Epigenetic Signalling (PPES), Department of Biomedical Sciences, University of Antwerp, Antwerp, Belgium

**Keywords:** NLRC4 inflammasome, inflammasomopathies, autoinflammation, microbiota, germfree mice, *Citrobacter rodentium*, *Salmonella* Typhimurium

## Abstract

**Background:**

Autoinflammation with infantile enterocolitis (AIFEC) is an often fatal disease caused by gain-of-function mutations in the NLRC4 inflammasome. This inflammasomopathy is characterized by macrophage activation syndrome (MAS)-like episodes as well as neonatal-onset enterocolitis. Although elevated IL-18 levels were suggested to take part in driving AIFEC pathology, the triggers for IL-18 production and its ensuing pathogenic effects in these patients are incompletely understood.

**Methods:**

Here, we developed and characterized a novel genetic mouse model expressing a murine version of the AIFEC-associated NLRC4^V341A^ mutation from its endogenous *Nlrc4* genomic locus.

**Results:**

NLRC4^V341A^ expression in mice recapitulated increased circulating IL-18 levels as observed in AIFEC patients. Housing NLRC4^V341A^-expressing mice in germfree (GF) conditions showed that these systemic IL-18 levels were independent of the microbiota, and unmasked an additional IL-18-inducing effect of NLRC4^V341A^ expression in the intestines. Remarkably, elevated IL-18 levels did not provoke detectable intestinal pathologies in NLRC4^V341A^-expressing mice, even not upon genetically ablating IL-18 binding protein (IL-18BP), which is an endogenous IL-18 inhibitor that has been used therapeutically in AIFEC. In addition, NLRC4^V341A^ expression did not alter susceptibility to the NLRC4-activating gastrointestinal pathogens *Salmonella* Typhimurium and *Citrobacter rodentium*.

**Conclusion:**

As observed in AIFEC patients, mice expressing a murine NLRC4^V341A^ mutant show elevated systemic IL-18 levels, suggesting that the molecular mechanisms by which this NLRC4^V341A^ mutant induces excessive IL-18 production are conserved between humans and mice. However, while our GF and infection experiments argue against a role for commensal or pathogenic bacteria, identifying the triggers and mechanisms that synergize with IL-18 to drive NLRC4^V341A^-associated pathologies will require further research in this NLRC4^V341A^ mouse model.

## 1 Introduction

Inflammasomes are multiprotein complexes that serve as a platform for inflammatory caspase activation. During canonical inflammasome activation, detection of pathogen-associated or danger-associated molecular patterns (PAMPs/DAMPs) by cytosolic sensor proteins leads to recruitment and proteolytic activation of caspase-1. Subsequently, activated caspase-1 proteolytically matures the proinflammatory cytokines pro-IL-1β and pro-IL-18, and cleaves Gasdermin D (GSDMD). The resulting N-terminal fragment of GSDMD is a pore forming protein that facilitates pyroptosis, a lytic form of cell death that allows the release of the mature IL-1β and IL-18 cytokines (1). Among various sensor proteins capable of inducing this inflammasome response, NLRC4 is activated by cytosolic flagellin or by components of the Type 3 secretion system (T3SS), which endows it with a pivotal role in antimicrobial defense against a wide range of bacterial pathogens such as *Pseudomonas aeruginosa* and *Salmonella* Typhimurium (2).

Besides its critical role in host defense against bacterial infections, NLRC4 was found to drive the development of a spectrum of autoinflammatory diseases (AIDs), collectively referred to as NLRC4-associated AIDs (NLRC4-AIDs) or NLRC4 inflammasomopathies (3). Several gain-of-function mutations in human NLRC4 such as the V341A (4, 5), T337S (6), T337N (7) and S171F (8) substitutions were described to cause such NLRC4-AIDs. Each of these NLRC4 mutations cause severe/recurrent macrophage activation syndrome (MAS)-like episodes characterized by elevated IL-18 serum levels (3). In addition, the NLRC4-AIDs caused by V341A (4, 5), T337S (6), T337N (7) or S171F (8) mutations exhibit neonatal-onset enterocolitis and are therefore also referred to as autoinflammation with infantile enterocolitis (AIFEC) (3). The first onset of AIFEC commonly occurs during infancy and leads to a potentially lethal disease. Based on NLRC4 crystal structure analysis, AIFEC-associated mutations are proposed to destabilize an auto-inhibitory mechanism of NLRC4, which might lead to ligand-independent spontaneous NLRC4 inflammasome activation (3, 4, 6, 8–11). However, possible triggers of AIFEC flares and the pathogenic mechanisms at work in AIFEC patients remain incompletely understood.

Here, we generated and characterized a knock-in mouse model of the AIFEC-associated NLRC4^V341A^ mutation during both homeostasis and infection. Our data show that murine NLRC4^V341A^ expression increases IL-18 levels in circulation as well as in the colon. However, elevated IL-18 levels in NLRC4^V341A^-expressing mice did not induce intestinal pathology and did not impact on gastrointestinal infections with NLRC4-activating pathogens. Interestingly, NLRC4^V341A^ induced IL-18 expression independently of the gut microbiota. Together, these observations argue against a role for commensal or pathogenic bacteria in driving NLRC4^V341A^-associated intestinal pathology, but further research will be needed to identify the actual triggers mediating NLRC4^V341A^-associated AIFEC.

## 2 Materials and methods

### 2.1 Mice

**Figure 1:**
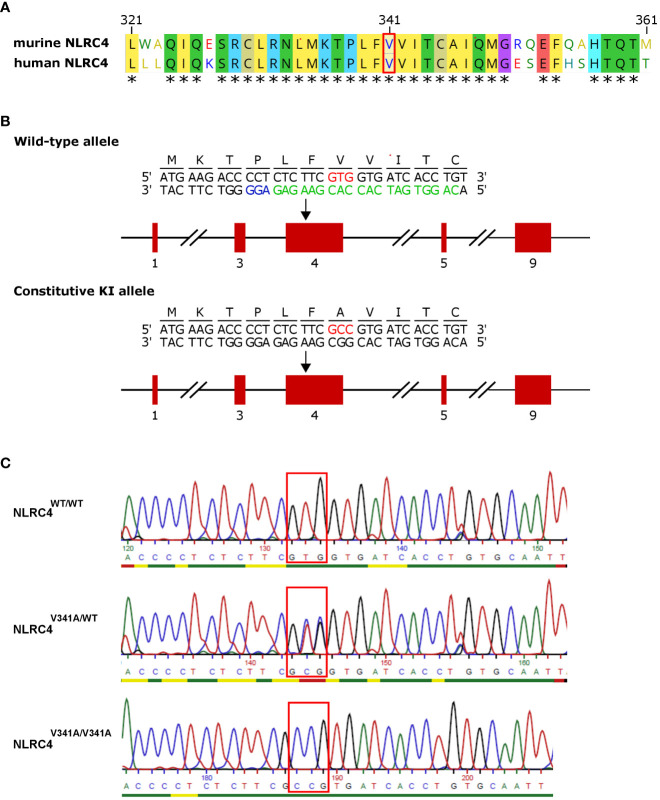
Generation of NLRC4^V341A^-expressing mice. **(A)** Alignment of amino acids 321-361 of human and murine NLRC4. **(B)** *Nlrc4* targeting scheme for introducing the V341A mutation. Red boxes are exons; the red codon is the V341A substitution; and in green and blue are the gRNA and the NGG target used for CrisprCas9 editing. **(C)** Genotyping sequencing example of resulting mice. * = amino acid residues conserved in humans and mice.

NLRC4^V341A/V341A^ mice were generated by Cyagen Biosciences by CRISPR/Cas9-mediated genome editing of the V341-encoding GTG codon to an A341-encoding GCC codon in exon 4 of the endogenous *NLRC4* gene of C57BL/6 mice (see also **Figure 1**). Briefly, C57BL/6 zygotes were co-injected with a vector encoding Cas9 as well as the gRNA 5’-CAGGTGATCACCACGAAGAG-3’ and with the 5’-tgtgggcccaaatccaggagtccaggtgcctgagaaatctgatgaagacccctctcttcGCCgtgatcacctgtgcaattcagatgggcagacaggaattccaagctcacacccaaaccatg-3’oligo donor for homology-directed repair. Pups were genotyped for the mutated GCC codon by sequencing a 361bp PCR amplicon around the mutated codon. Two founder animals carrying the correct GCC mutation and no other mutations within the amplicon were crossed to C57BL/6J mice and then intercrossed to obtain homozygous Nlrc4^V341A/V341A^ mice. Upon observing that Nlrc4^V341A/V341A^ offspring from both founders were born in Mendelian ratios and appeared healthy, all further analyses were performed on progeny from one founder animal. Il18bp^tm1.1(KOMP)Vlcg^ (IL-18BP^-/-^) mice were generated by the Knockout Mouse Project Repository (University of California, Davis, Davis, CA) on C57BL/6N background by the Velocigene approach, in which the entire coding region of the *Il18bp* gene was replaced by a LacZ gene and a LoxP-flanked Neomycin cassette (12). Specific pathogen free (SPF) mice were housed in individually ventilated cages in the animal facility of the IRC-VIB. Germfree (GF) Nlrc4^V341A/V341A^ mice were generated by transfer of Nlrc4^V341A/+^ embryo’s in GF foster mothers that had been mated with GF vasectomized males, after which the resulting GF Nlrc4^V341A/+^ mice were intercrossed to generate GF Nlrc4^+/+^ and Nlrc4^V341A/V341A^ littermates. GF mice were generated at the Axenic/Gnoto Facility of the Instituto Gulbenkian de Ciência (Lisbon, Portugal) in the context of an INFRAFRONTIER2020 project, and were then transfered to a GF facility at Ghent University where they were housed in positive-pressure flexible film isolators (North Kent Plastics). Food and water were provided ad libitum. All animal experiments were performed according to institutionally approved protocols according to national (Belgian Laws 14/08/1986 and 22/12/2003, Belgian Royal Decree 06/04/2010) and European (EU Directives 2010/63/EU, 86/609/EEG) animal regulations. Animal protocols were reviewed and approved by the Ethical Committee Animal Experimentation VIB site Ghent - Ghent University - Faculty of Sciences (permit number LA1400091) with approval IDs 2018-032 and 2019-072. All necessary efforts were made to minimize suffering of the animals.

### 2.2 Oral *S.* Typhimurium infection

Age- and sex-matched mice were infected by oral gavage with 10^4^ or 10^5^ CFU of the SL1344 *S.* Typhimurium strain administered in a 100 μl inoculum in the logarithmic phase of proliferation after starvation of 4h. Overall susceptibility to the infection was evaluated by survival.

### 2.3 Gastrointestinal *Citrobacter rodentium* infection

Age- and sex-matched mice were infected by oral gavage with 5 x 10^9^ CFU of the nalidixic acid (NAL) resistant ICC169 *C. rodentium* strain administered in a 200 μl inoculum in the logarithmic phase of proliferation, as described (13). Enumeration of *C. rodentium* loads was performed by plating stool samples and colon tissues collected at the indicated time points after infection, on selective Luria-Bertani (LB) agar containing 50 μg/ml NAL. Colony forming units (CFUs) were normalized to the weight of the sample. Mice that did not surpass the threshold of 1 x 10^5^ CFU/g feces at 7 days post-infection (dpi) were considered not successfully infected and were removed from the experiment.

### 2.4 Stimulation of bone marrow derived macrophages

Primary macrophages were generated from bone marrow cells flushed from femur and tibia. Cells were differentiated to bone marrow derived macrophages (BMDMs) by culturing them in Iscove’s modified Dulbecco’s medium (IMDM, Lonza) with 10% (v/v) heat-inactivated FBS, 30% (v/v) L929 cell-conditioned medium, 1% Gibco non-essential amino acids, penicillin (100 U/ml) and streptomycin (100 mg/ml) for 6 days in 37°C in the presence of 5% CO_2_. After differentiation, medium was aspirated and the cells were scraped in IMDM supplemented with 10% FBS and 1% Gibco non-essential amino acids. For specific experiments, 10^6^ cells were seeded in 0.5 ml culture medium per well in 12-well plates and were incubated overnight at 37˚ C and 5% CO_2_. On day 7, FlaTox stimulations (1 µg/ml, 2h) and *S.* Typhimurium infections (MOI1, 3h) were performed on LPS-primed (1 µg/ml, 5h) or unprimed BMDMs. At 2h post *S.* Typhimurium infection 100 µg/ml gentamycin was added followed by 1h incubation. After incubation at 37°C and 5% CO_2_ samples were collected.

### 2.5 Cell death analysis

Lytic cell death was evaluated by detection of LDH released in the cell culture supernatant by LDH assay (Promega) according to manufacturer’s instructions. The data were plotted as percentage of total cell death as determined by a 100% cell death control from Triton X-100 treated wells.

### 2.6 Cytokine measurements

Tissue samples were weighed and were homogenized in PBS with protease inhibitors, after which lysis was completed by addition of lysis buffer (20 mM Tris HCl (pH 7.4), 200 mM NaCl, 1% Nonidet P-40) and incubation for 20 minutes on ice. Full speed centrifugation for 30 minutes cleared the homogenate and supernatant was used for further analysis. Mouse cytokines in cell culture supernatants, serum and tissue homogenates were determined by magnetic bead-based multiplex assay using Luminex technology (Bio-Rad, Hercules, CA, USA) according to the manufacturer’s instructions. Cytokines from tissue homogenates were normalized to weight of tissue, while cytokines from cell culture supernatants and serum were expressed as concentration per ml of cell culture medium or serum.

### 2.7 Western blot analyses

Cells and culture supernatants, colon or ileum intestinal epithelial cells (IECs), or whole colon or ileum homogenates, were incubated with cell lysis buffer (20 mM Tris HCl (pH 7.4), 200 mM NaCl, 1% Nonidet P-40) and denatured in Laemlli buffer by boiling for 10 min. Proteins were separated by SDS-PAGE electrophoresis (Thermo Scientific) after which proteins were transferred to membranes using turbo (7 min) blotting. Blocking and antibody incubation were performed in PBS supplemented with 0.05% or 0.2% Tween20 (vol/vol) and 3% non-fat dry milk. The membranes were incubated overnight at 4˚ C with primary antibodies against caspase-1 (1:1000; Adipogen, AG-20B-0042-C100), IL-1β (1:2000; GeneTex, GTX74034), GSDMD (1:1000, Abcam, ab209845) and IL-18 (1:1000, Biovision, 5180R-100). After washing, membranes were incubated with HRP-conjugated anti-mouse, anti-rabbit or anti-goat antibodies (1:5000; Jackson ImmunoResearch Laboratories, 115-035-146, 111-035-144 and 305-035-003) or were incubated with the directly labeled primary antibody β-Actin-HRP (1:10000; Santa Cruz) for up to 3h. Proteins of interest were detected by the enhanced SuperSignal West Pico Chemiluminescent Substrate (Thermo Scientific).

### 2.8 Histology

Colon and ileum tissues were fixed in 4% paraformaldehyde, embedded in paraffin, and cut in 4 µm sections. For histopathological analysis hematoxylin and eosin staining were performed according to standard protocols. Histological crypt length quantifications were performed in a blinded fashion using Image-J-win4. Cell death was evaluated on paraffin sections by TUNEL staining (*in situ* cell death detection kit, TMR red, Roche) performed according to the manufacturer’s instructions. For immunohistochemical Ki67 staining paraffin sections were rehydrated and heat-induced antigen retrieval was performed in Antigen Unmasking Solution, Citric Acid Based (Vector Laboratories). Endogenous peroxidase activity was blocked by incubating the slides in methanol containing 3% H_2_O_2_. Sections were then incubated overnight with primary antibody for Ki67 (1/1000, Cell Signalling, D3B5) in PBS containing 10% goat serum. Biotinylated secondary antibody was purchased from Dako (E0432). Stainings were visualized with Vectastain ELITE ABC Kit and DAB substrate (ImmPACT DAB Substrate kit, Peroxidase, Vector Laboratories), after which sections were counterstained with hematoxylin. Incubation times with DAB substrate were equal for all samples. For AB-PAS staining paraffin sections were rehydrated, stained for 20 min with Alcian blue. This was followed by 15 min treatment with 1% periodic acid and 15 min treatment with Shiff’s reagent. After washing with running tap water for 10 min, nuclei were stained with Mayer’s hematoxylin for 20 sec. Finally, sections were dehydrated and mounted with depex. For immunofluorescent Lysozyme staining paraffin sections were rehydrated and heat-induced antigen retrieval was performed in Antigen Unmasking Solution, Citric Acid Based (Vector Laboratories). Endogenous peroxidase activity was blocked by incubating the slides in methanol containing 3% H_2_O_2_. Sections were incubated overnight with anti-lysozyme primary antibody (1/700, DAKO, A0099) in PBS containing 0.2% goat serum, 0.5% fish skin gelatin and 2% BSA, followed by one-hour incubation with a secondary antibody labeled with Dylight-488 (1/500, Thermofisher, 35553) and nuclei staining with DAPI (1/1000, Thermofisher, D21490) for 10min in Prolong Gold anti-fade. All pictures were taken with a high content screening microscope (Zeiss AxioScan) at the same exposure and intensity settings.

### 2.9 Isolation of IECs

Ileum and colon were dissected and flushed with PBS to remove fecal content. The tissue was turned inside-out, washed with PBS and incubated in HBSS containing 2 mM EDTA for 30 min at 37°C shaking. Afterwards tissue was removed and cells were collected by centrifugation at 3000 rpm for 5 min, followed by washing of the cells with PBS by centrifugation at 5000 rpm for 5 min. Cells were lysed in cell lysis buffer (20 mM Tris HCl (pH 7.4), 200 mM NaCl, 1% Nonidet P-40) for Western Blot analysis or kept in TRIsure (Bioline) until further isolation of RNA.

### 2.10 Quantitative real-time PCR

Total RNA was isolated using TRIsure reagent (Bioline) and the RNeasy^®^ Mini Kit (QIAGEN) according to manufacturer’s instructions. cDNA was synthesized using the iScript gDNA clear cDNA Synthesis kit (Biorad) according to the manufacturer’s instructions. cDNA was amplified on quantitative PCR in a total volume of 5 µl with SensiFAST SYBR^®^ No-ROX Kit (Bioline) and specific IL-18 primers (Fwd ACTTTGGCCGACTTCACTGTA, Rev CTTCACAGAGAGGGTCACAGC) on a LightCycler 480 (Roche). The reactions were performed in triplicates. Primer sequences reference genes: Gapdh (Fwd TGAAGCAGGCATCTGAGGG, Rev CGAAGGTGGAAGAGTGGGAG), Hprt1 (Fwd AGTGTTGGATACAGGCCAGAC, Rev CGTGATTCAAATCCCTGAAGT), Ubc (Fwd AGGTCAAACAGGAAGACAGACGTA, Rev TCACACCCAAGAACAAGCACA).

### 2.11 Statistics

All statistical analyses were performed using GraphPad Prism version 9.0. For mouse survival curves, statistical significance was determined by log-rank Mantel-Cox test. Other data were analyzed by applying either unpaired two-sided student *t*-tests or unpaired two-sided Mann-Whitney tests in case of not normal distribution of the values. Data are shown as means of biological replicates with SD as indicated in figure legends. Statistical results are indicated as ns not significant; *p < 0.05; **p < 0.01 or ***p < 0.001.

## 3 Results

### 3.1 NLRC4^V341A^ expression elevates IL-18 in circulation but does not provoke intestinal inflammation in mice

The V341A mutation of human *NLRC4* was reported to cause AIFEC characterized by neonatal-onset enterocolitis, periodic fevers, and fatal or near-fatal episodes of autoinflammation in four individuals from two unrelated pedigrees (4, 5). To better understand the pathogenic mechanisms involved in these NLRC4^V341A^-expressing AIFEC patients, we created mice expressing a V341A variant of the murine NLRC4 protein, as the region around the V341 residue is conserved in humans and mice (**Figure 1A**). For this purpose, we introduced a codon encoding the V341A substitution in the endogenous *Nlrc4* gene of C57BL/6 mice by CRISPR/Cas9-mediated genome editing (**Figures 1B, C**). Both NLRC4^V341A/WT^ and NLRC4^V341A/V341A^ mice were born in Mendelian ratios and aged indistinguishably from their NLRC4^WT/WT^ littermates. Therefore, to maximize potential pathologic effects of NLRC4^V341A^ expression, we proceeded with analyzing inflammatory parameters in homozygous NLRC4^V341A/V341A^ mice as compared to their NLRC4^WT/WT^ littermates.

**Figure 2:**
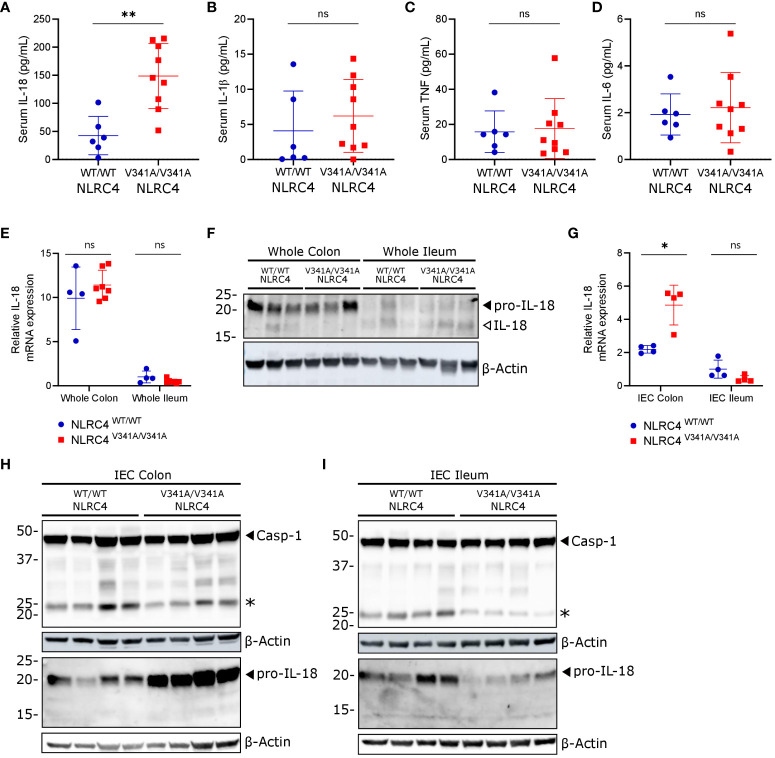
NLRC4^V341A^ expression provokes elevated IL-18 circulation and increased pro-IL-18 expression in colon IECs. **(A–D)** Serum **(A)** IL-18, **(B)** IL-1β, **(C)** TNF and **(D)** IL-6 levels in age- and sex-matched NLRC4^V341A/V341A^ and NLRC4^WT/WT^ littermates. **(E–I)** IL-18 **(E, G)** mRNA and **(F, H, I)** protein expression in **(E, F)** whole colon and ileum or in **(G–I)** colon and ileum IECs of NLRC4^V341A/V341A^ and NLRC4^WT/WT^ littermates. Data in **(A–E, G)** represent individual mice and their means +/- SD. In **(F, H, I)** every lane represents a lysate from a different mouse, and * indicates an unspecific signal in **(H, I)**. * p<0.05, ** p<0.01, ns, not significant.

AIFEC patients show chronically elevated serum IL-18 levels that are thought to originate from increased NLRC4 inflammasome activity (4–7). Therefore, to determine whether NLRC4^V341A^-expressing mice displayed a similar IL-18-dominant serum signature, we measured the inflammasome-dependent IL-18 and IL-1β as well as the inflammasome-independent IL-6 and TNF cytokines in serum of NLRC4^V341A/V341A^ and NLRC4^WT/WT^ mice. NLRC4^V341A/V341A^ mice showed significantly higher circulating IL-18 levels than NLRC4^WT/WT^ littermates (**Figure 2A**), while IL-1β, IL-6 and TNF serum levels did not differ between these genotypes (**Figures 2B–D**). Thus, as observed in AIFEC patients (4–7), NLRC4^V341A^ expression in mice induces a specific increase in circulating IL-18 levels.

A previously described genetic mouse model expressing an NLRC4^T337S^ mutant showed that IL-18 production in these mice mainly originated from IECs (14). We therefore investigated whether the gastrointestinal tract was the source of increased circulating IL-18 levels in NLRC4^V341A/V341A^ mice. Whole tissue qPCR analyses showed no differences in pro-IL-18 mRNA expression in ileum or colon of NLRC4^V341A/V341A^ mice, while these analyses did show that IL-18 mRNA expression was higher in colon compared to ileum in both genotypes (**Figure 2E**). In accordance, pro-IL-18 protein was mainly detectable in colon lysates, but NLRC4^V341A/V341A^ mice did not show different pro-IL-18 levels compared to NLRC4^WT/WT^ mice when analyzing complete ileum or colon lysates (**Figure 2F**). In addition, these protein analyses on complete tissue lysates only detected low levels of mature IL-18 in either ileum or colon, and did not reveal clear effects of NLRC4^V341A^ expression on IL-18 maturation (**Figure 2F**). Next, given their high IL-18 expression levels (14–16), we analyzed IL-18 production specifically in IECs of ileum and colon of NLRC4^V341A/V341A^ mice. Interestingly, whereas IECs isolated from ileum showed no differences in pro-IL-18 mRNA expression, colon IECs from NLRC4^V341A/V341A^ mice showed higher pro-IL-18 mRNA expression than colon IECs from NLRC4^WT/WT^ mice (**Figure 2G**). Accordingly, immunoblotting analyses showed that NLRC4^V341A^ expression increased pro-IL-18 protein levels in colon but not in ileum IECs (**Figures 2H, I**). However, these immunoblotting analyses did not allow detecting mature IL-18 in IECs. In addition, cleavage of caspase-1 to its active p20 subunit was undetectable in colon or ileum IECs of naïve NLRC4^V341A/V341A^ mice (**Figures 2H, I**). Overall, these observations indicate that NLRC4^V341A^ expression increases pro-IL-18 mRNA as well as protein levels specifically in colonic IECs, but inflammasome activation leading to IL-18 maturation could not be detected in these cells under homeostatic conditions. Therefore, although our observations suggest that NLRC4^V341A^-expressing colon IECs have a higher capacity to produce mature IL-18, it remains unclear whether the elevated circulating IL-18 levels in these mice derive from the intestines.

**Figure 3:**
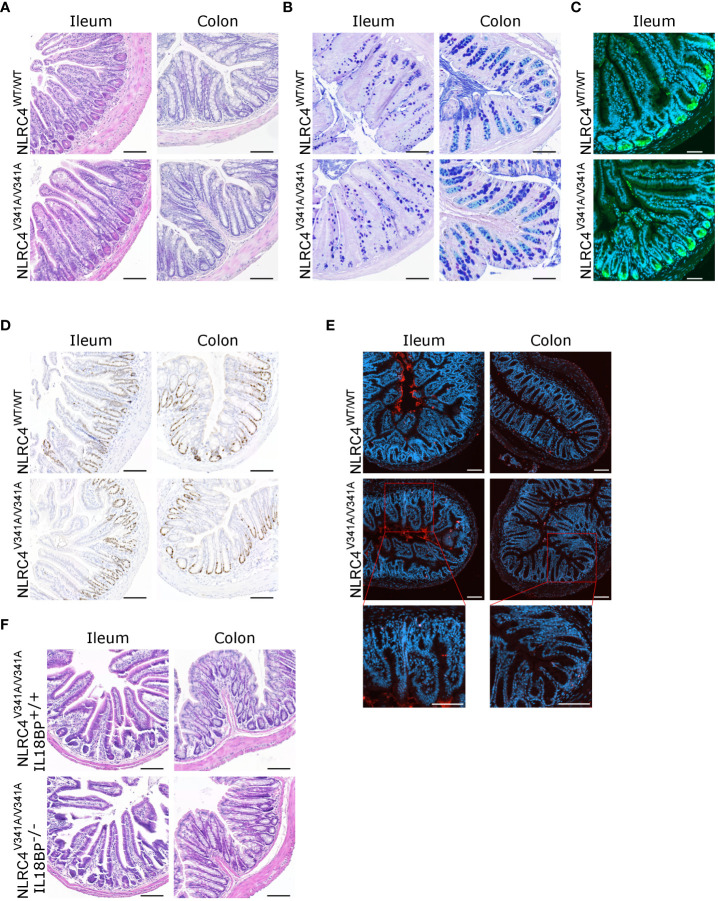
Nlrc4^V341A^ expression does not affect intestinal homeostasis even in the absence of IL-18BP. **(A–E)** Representative **(A)** H&E staining, **(B)** AB-PAS staining, **(C)** Lysozyme staining, **(D)** Ki67 staining and **(E)** TUNEL staining on colon and/or ileum of NLRC4^V341A/V341A^ and NLRC4^WT/WT^ littermates. **(F)** Representative ileum and colon H&E staining of age- and sex-matched NLRC4^V341A/V341A^IL-18BP^-/-^ and NLRC4^V341A/V341A^IL-18BP^+/+^ littermates. Scale bars 100 µm.

Intestinal biopsies and autopsy specimens from NLRC4^V341A^-expressing AIFEC patients showed mixed immune cell infiltration, villous blunting with tissue edema, and tissue autolysis (4, 5). Given this remarkable intestinal pathology and the increased pro-IL-18 production by colonic IECs in NLRC4^V341A/V341A^ mice, we performed histopathological analyses on ileum and colon of these mice. H&E staining revealed no abnormal immune cell infiltration or structural differences in both ileum or colon of NLRC4^V341A/V341A^ mice (**Figure 3A**). Furthermore, normal numbers of goblet cells and Paneth cells were observed in NLRC4^V341A/V341A^ mice as assessed by Alcian Blue-Periodic acid-Schiff (AB-PAS) staining and Lysozyme immunostaining, respectively (**Figures 3B, C**). Finally, NLRC4^V341A/V341A^ mice showed normal rates of intestinal epithelial proliferation (**Figure 3D**), and a TUNEL staining revealed no increased cell death in ileum or colon of NLRC4^V341A/V341A^ mice (**Figure 3E**). Taken together, these analyses suggest that the increased pro-IL-18 levels in colon IECs do not give rise to sufficient IL-18 activity to induce histopathological changes in the intestine of NLRC4^V341A/V341A^ mice under homeostatic conditions.

IL-18 Binding Protein (IL-18BP)-mediated sequestering of IL-18 prevents the mature cytokine from binding with its receptor (17–19). To investigate whether this might provide a possible explanation for the lack of intestinal inflammation in NLRC4^V341A/V341A^ mice, we generated NLRC4^V341A/V341A^ mice in an IL-18BP-deficient background. However, the absence of IL-18BP did not provoke histopathology in ileum or colon of these NLRC4^V341A/V341A^IL-18BP^-/-^ mice (**Figure 3F**). This indicates that the absence of intestinal pathology in NLRC4^V341A/V341A^ mice did not result from IL-18 neutralization by IL-18BP. Overall, the above analyses show that despite their increased IL-18 circulation, naïve NLRC4^V341A/V341A^ mice do not display gastrointestinal pathologies even in the absence of the endogenous IL-18 antagonist IL-18BP.

### 3.2 NLRC4^V341A^ expression does not alter host defense against infections with NLRC4-triggering gastrointestinal pathogens

**Figure 4:**
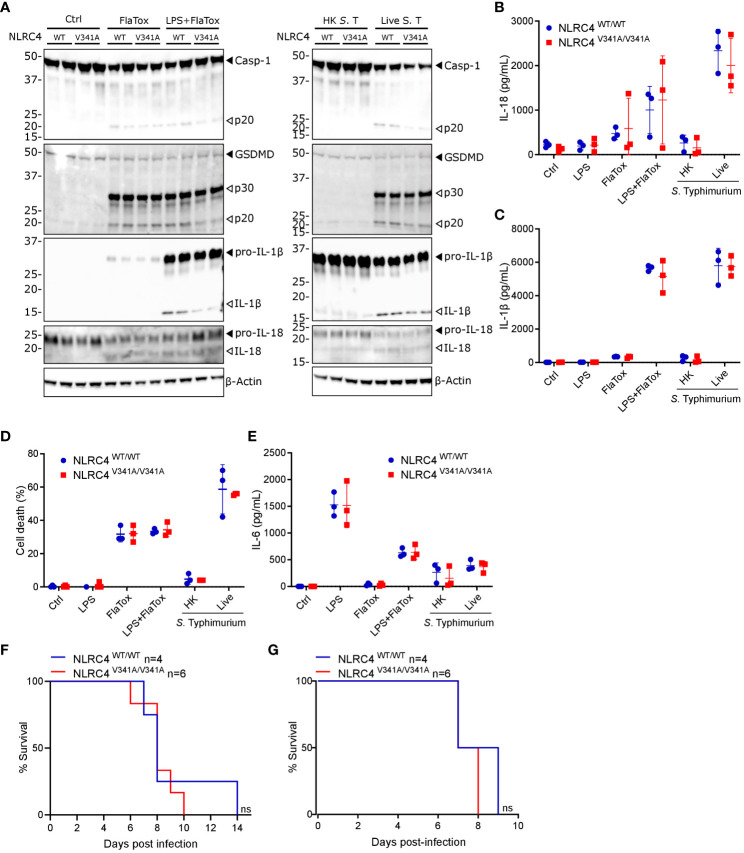
Nlrc4^V341A^ expression does not impact on inflammasome activation by NLRC4 stimuli in BMDMs or on *S.* Typhimurium susceptibility. **(A–E)** NLRC4^WT/WT^ and NLRC4^V341A/V341A^ BMDMs were left untreated (Ctrl), were primed or not for 5h with LPS, were treated with FlaTox for 2h, or were infected with heat-killed (HK) or live *S.* Typhimurium (*S.* T) at MOI 1 for 3h, as indicated. **(A)** Western blot analysis of caspase-1, GSDMD, IL-1β and IL-18. **(B–E)** Culture supernatant levels of **(B)** IL-18, **(C)** IL-1β, **(D)** Lactate Dehydrogenase (LDH), and **(E)** IL-6. **(F, G)** Survival analyses of age-matched **(F)** female or **(G)** male NLRC4^V341A/V341A^ and NLRC4^WT/WT^ littermates infected by oral gavage with **(F)** 10^5^ CFU or **(G)** 10^4^ CFU *S.* Typhimurium. In **(A)** every lane represents BMDMs from a different mouse. Data in **(B–E)** are means +/- SD of biological triplicates. ns, not significant.

As our above analyses showed that elevated IL-18 circulation in naïve NLRC4^V341A/V341A^ mice was not associated with gastrointestinal pathologies, we next evaluated the response of these mice to gastrointestinal infections. Indeed, NLRC4 plays important roles in host defense against a wide range of NLRC4-activating bacterial pathogens, for which the enteropathogen *Salmonella* Typhimurium is a prototypical example (20). To assess whether NLRC4^V341A^ intrinsically alters NLRC4-mediated inflammasome activation, we first evaluated NLRC4^V341A^ responses in bone-marrow-derived macrophages (BMDMs). For this purpose, we treated BMDMs with FlaTox to deliver flagellin to the cytosol (21, 22), and we infected BMDMs with *S.* Typhimurium. Upon both of these NLRC4-activating triggers, NLRC4^V341A/V341A^ and NLRC4^WT/WT^ BMDMs showed similar caspase-1, GSDMD and IL-18 cleavage (**Figure 4A**), and showed no significant differences in IL-18 secretion (**Figure 4B**). In addition, although LPS-primed NLRC4^V341A/V341A^ BMDMs showed a slight reduction in IL-1β cleavage compared to NLRC4^WT/WT^ BMDMs upon FlaTox stimulation (**Figure 4A**), FlaTox- as well as *S.* Typhimurium-induced IL-1β secretion was equal between these genotypes (**Figure 4C**). In line with these cytokine observations, NLRC4^V341A/V341A^ and NLRC4^WT/WT^ BMDMs died to a similar extent upon FlaTox stimulation or *S.* Typhimurium infection (**Figure 4D**). Notably, in each of these inflammasome activation, cytokine and cell death analyses, untreated NLRC4^V341A/V341A^ and NLRC4^WT/WT^ BMDMs showed no baseline differences (**Figures 4A–D**). Taken together, these data indicate that NLRC4^V341A^ expression does not alter either baseline or stimulated NLRC4 inflammasome activity in BMDMs. Furthermore, baseline as well as LPS- and *S.* Typhimurium-induced IL-6 production was similar between NLRC4^V341A/V341A^ and NLRC4^WT/WT^ BMDMs (**Figure 4E**), suggesting that also NF-κB transcriptional effects in BMDMs were not affected by NLRC4^V341A^ expression. Overall, these data show that NLRC4^V341A^ expression does not alter *S.* Typhimurium responses in BMDMs. Next, to extend these analyses to a physiological infectious context, we infected NLRC4^V341A/V341A^ and NLRC4^WT/WT^ mice orally with *S.* Typhimurium as a model of lethal typhoid fever caused by systemic pathogen dissemination from the gastrointestinal tract (23, 24). However, neither female nor male NLRC4^V341A/V341A^ mice showed differences in survival upon *S.* Typhimurium infection as compared to their NLRC4^WT/WT^ littermates (**Figures 4F, G**). This observation shows that NLRC4^V341A^ expression does not influence the overall host response against *S.* Typhimurium induced typhoid fever-like disease, which together with our BMDM observations argues against NLRC4^V341A^ critically altering NLRC4 inflammasome responses during *S.* Typhimurium infection.

**Figure 5:**
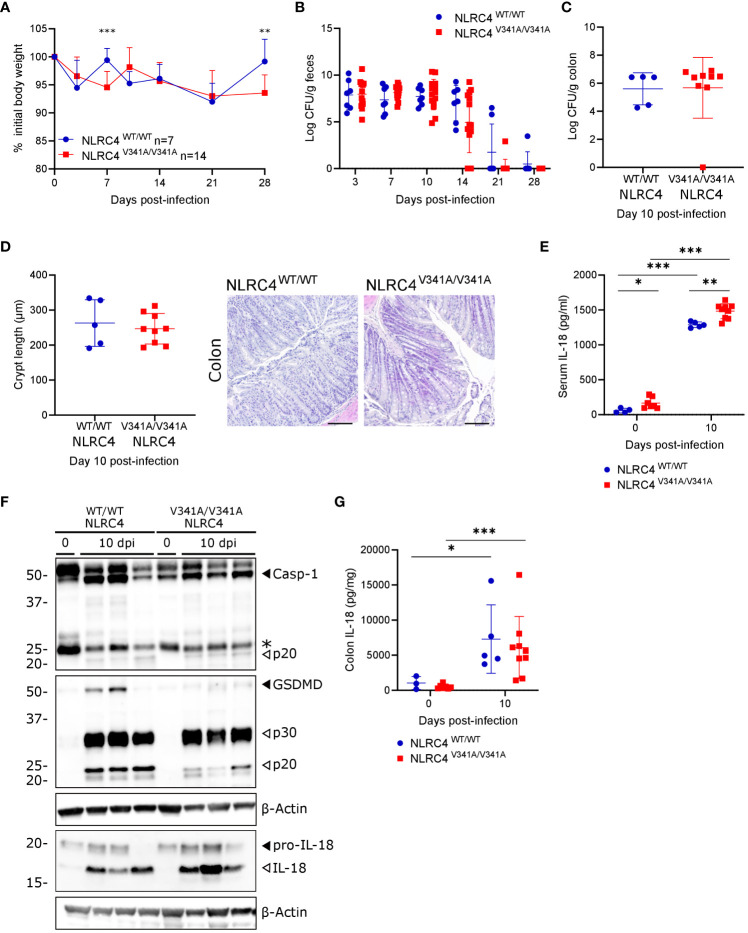
Nlrc4^V341A^ expression does not impact on the host defense against a gastrointestinal *C. rodentium* infection. **(A–G)** Age- and sex-matched NLRC4^V341A/V341A^ and NLRC4^WT/WT^ littermates were not infected or infected by oral gavage with 5x10^9^ CFU *C*. *rodentium* and analyzed at indicated days post-infection (dpi). **(A)** Weight change, **(B)** fecal *C*. *rodentium* loads, **(C)** colonic *C*. *rodentium* loads, **(D)** representative colon H&E staining and colon crypt length measurements, **(E)** serum IL-18 levels, **(F)** Western blot analyses on whole colon lysates, and **(G)** whole colon IL-18 levels. Data in **(A)** represent means + SD; data in **(B–E, G)** represent individual mice and their means +/- SD. In **(F)** every lane represents a whole colon lysate from a different mouse, and * indicates an unspecific signal. Scale bars **(D)** 100 µm. * p<0.05, ** p<0.01, *** p<0.001.

As a second gastrointestinal infection model we inoculated NLRC4^V341A/V341A^ mice by oral gavage with *C. rodentium*, a murine enteropathogen used to mimic enteropathogenic and enterohaemorrhagic *Escherichia coli* infections in humans (25). Notably, NLRC4 inflammasome activation was demonstrated to participate in host defense against *C. rodentium* by acting in non-hematopoietic cells (26), thereby providing an opportunity to assess potential *in vivo* effects of epithelial NLRC4^V341A^ expression. During *C. rodentium* infection, NLRC4^V341A/V341A^ mice did not display consistent body weight changes compared to NLRC4^WT/WT^ littermates (**Figure 5A**), and these mice cleared the infection with similar kinetics as NLRC4^WT/WT^ littermates (**Figure 5B**). Furthermore, NLRC4^V341A/V341A^ mice did not show differences in colonic *C. rodentium* loads at the peak of infection [10 days post-infection (dpi)] (**Figure 5C**). In addition, whereas transmissible colonic crypt hyperplasia is a characteristic histopathological feature of *C. rodentium* colitis, NLRC4^V341A/V341A^ and NLRC4^WT/WT^ mice showed comparable crypt lengths at 10 dpi (**Figure 5D**). Overall, these observations indicate no obvious influence of NLRC4^V341A^ expression on the host defense against a gastrointestinal *C. rodentium* infection. However, *C. rodentium* further increased serum IL-18 levels in NLRC4^V341A/V341A^ mice and, as observed in uninfected conditions, *C. rodentium-*infected NLRC4^V341A/V341A^ mice showed higher IL-18 levels in the serum compared to their NLRC4^WT/WT^ littermates (**Figure 5E**). Moreover, in contrast to our observations in uninfected mice, whole colon lysates of *C. rodentium-*infected mice showed the presence of mature IL-18 as well as cleaved caspase-1 and GSDMD, indicating that this infection induced inflammasome activation in the colon (**Figure 5F**). Nevertheless, these Western blotting analyses showed similar levels of colonic inflammasome activation in *C. rodentium-*infected NLRC4^V341A/V341A^ and NLRC4^WT/WT^ mice (**Figure 5F**). An IL-18 ELISA approach to more specifically quantify mature IL-18 levels confirmed this semi-quantitative Western blotting observation, as whole colon lysates of NLRC4^V341A/V341A^ and NLRC4^WT/WT^ mice showed equal IL-18 upregulation (**Figure 5G**). Together, these observations suggest that NLRC4^V341A^ expression does not induce excessive inflammasome responses during a *C. rodentium* infection and thereby does not impact on the host response against this enteropathogen. Considering also our observations with *S.* Typhimurium, these *C. rodentium* observations argue that NLRC4^V341A^ expression does not influence the host response to NLRC4-triggering pathogens.

### 3.3 NLRC4^V341A^ increases systemic and intestinal IL-18 levels independently of the microbiota

**Figure 6:**
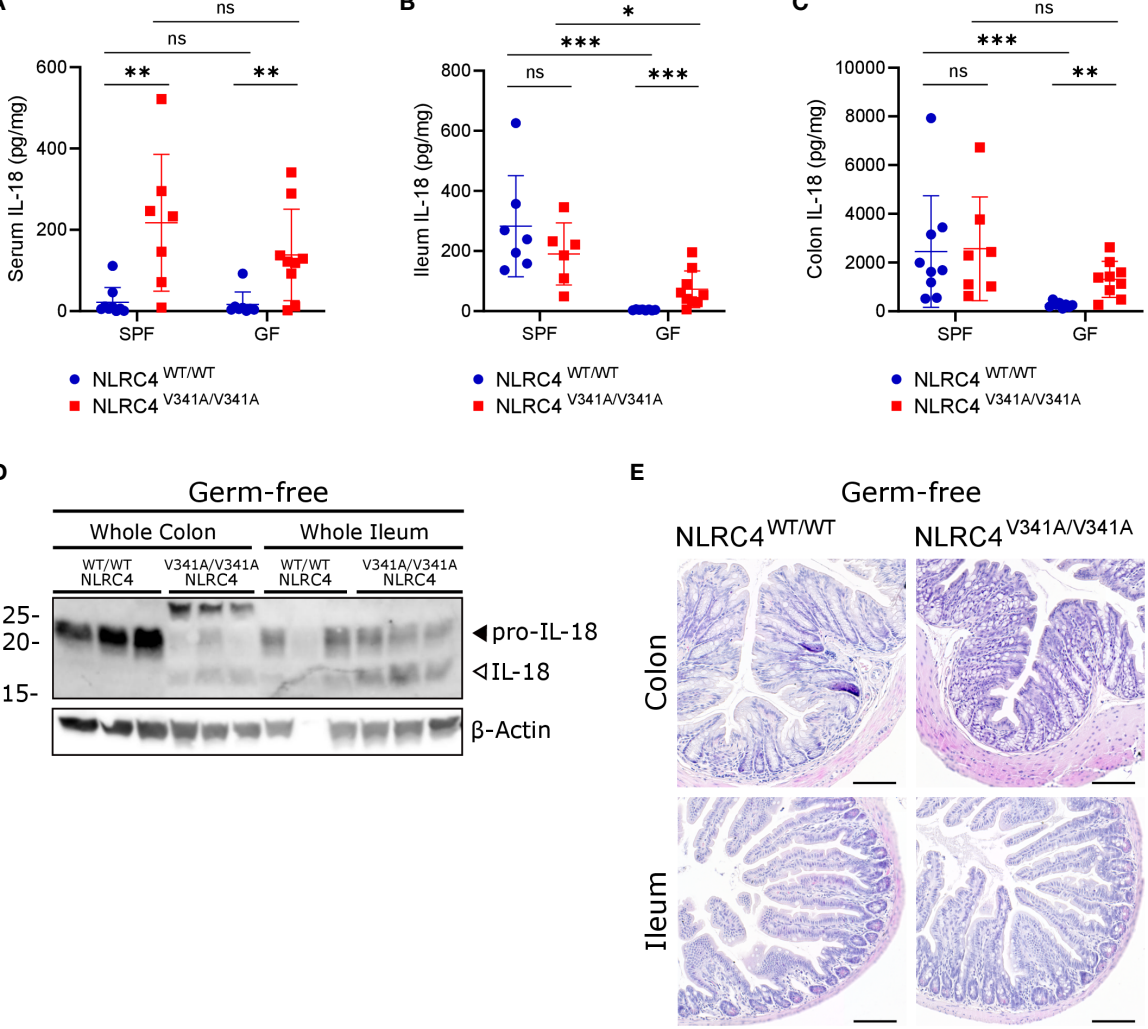
NLRC4^V341A^ expression increases systemic and intestinal IL-18 levels independently of the microbiota. **(A–E)** Age- and sex-matched NLRC4^V341A/V341A^ and NLRC4^WT/WT^ littermates, housed in either SPF or GF conditions as indicated, were analyzed for **(A)** IL-18 serum levels, **(B)** ileum IL-18 levels, **(C)** colon IL-18 levels, **(D)** IL-18 maturation in whole colon and ileum lysates, and **(E)** ileum and colon histopathology by H&E staining. Data in **(A–C)** represent individual mice and their means +/- SD. Every lane in **(D)** represents a whole ileum or colon lysate from a different mouse. Scale bars **(E)** 100 µm. * p<0.05, ** p<0.01, *** p<0.001, ns, not significant.

While our above observations argued against a role for pathogenic bacteria in exacerbating NLRC4^V341A^-induced IL-18 production, we next aimed to investigate whether commensal bacteria were responsible for the observed IL-18 production in NLRC4^V341A/V341A^ mice. For this purpose, we generated germfree (GF) NLRC4^V341A/V341A^ mice that are devoid of microbiota. Remarkably, GF NLRC4^V341A/V341A^ mice showed significantly higher IL-18 levels in circulation compared to GF NLRC4^WT/WT^ mice, similar as observed under specific pathogen free (SPF) conditions (**Figure 6A**). This showed that systemic IL-18 elevation in NLRC4^V341A/V341A^ mice was independent of the microbiota. Moreover, analyzing IL-18 levels in ileum and colon of GF NLRC4^V341A/V341A^ mice revealed that the absence of microbiota unmasked an IL-18-inducing effect of NLRC4^V341A^ in the intestine. Consistent with a role for the gut microbiota in inducing basal IL-18 production (27–30), ileal and colonic IL-18 production were abolished in GF NLRC4^WT/WT^ mice (**Figures 6B, C**). However, GF NLRC4^V341A/V341A^ mice showed increased IL-18 levels in both ileum and colon (**Figures 6B, C**), thereby demonstrating that NLRC4^V341A/V341A^ mice produce intestinal IL-18 independently of the microbiota. Consistent with these ELISA-based mature IL-18 measurements, western blot analyses showed increased IL-18 cleavage in both the colon and ileum of GF NLRC4^V341A/V341A^ mice compared to GF NLRC4^WT/WT^ mice (**Figure 6D**). This suggests that the microbiota-independent IL-18 production in GF NLRC4^V341A/V341A^ mice could be associated with NLRC4^V341A^-mediated inflammasome activation. However, despite the detectable elevations in intestinal mature IL-18 production in GF mice, like SPF NLRC4^V341A/V341A^ mice also GF NLRC4^V341A/V341A^ mice showed no obvious histopathology in both ileum or colon (**Figure 6E**). Taken together, these observations show that NLRC4^V341A^ expression increases intestinal as well as systemic IL-18 levels independently of the microbiota, while these elevated IL-18 levels do not suffice to provoke gastrointestinal pathology.

## 4 Discussion

Inflammasomopathies are autoinflammatory disorders caused by gain-of-function mutations in inflammasome sensor proteins. Among the various known inflammasomopathies, NLRC4-AIDs distinguish themselves from NLRP3-associated Cyropyrin-associated periodic syndromes (CAPS) and Pyrin-related Familial Mediterranean Fever (FMF) by the occurrence of MAS, elevated IL-18 production and the manifestation of potentially lethal infantile enterocolitis in the AIFEC subset of NLRC4-AID patients (3, 31, 32). In addition, while CAPS and FMF patients are treated effectively with IL-1-antagonizing therapeutics (33), an NLRC4^V341A^-expressing AIFEC patient was found to be refractory to anakinra treatment. However, supplementing anakinra treatment with recombinant human IL-18BP, which is a circulating decoy protein for IL-18, was able to resolve MAS parameters as well as enterocolitis in this one NLRC4^V341A^-expressing AIFEC patient (5). While this demonstrated a potential pathogenic contribution for IL-18 in some individuals expressing an NLRC4^V341A^ variant, the mechanisms underlying NLRC4^V341A^-associated IL-18 production and subsequent colitis development are not known.

In this study, we generated a genetic mouse model expressing a murine NLRC4^V341A^ variant from its endogenous genomic locus. Interestingly, homozygous NLRC4^V341A/V341A^ mice recapitulated elevated serum IL-18 levels as in AIFEC patients (4–7), although the IL-18 serum levels observed in NLRC4^V341A/V341A^ mice were much lower than observed in NLRC4^V341A^-expressing AIFEC patients (4, 5). This species difference could relate either to an intrinsically lower caspase-1 activating capacity of the murine NLRC4^V341A^ mutant, to less pro-IL-18 expression in mouse IECs as compared to human IECs, or to insufficient levels of NLRC4-activating stimuli in SPF-housed mice. However, regarding the latter possibility it should be noted that even *C. rodentium*-infected NLRC4^V341A/V341A^ mice showed tenfold less serum IL-18 than AIFEC patients, indicating that a pathogenic NLRC4 activator in the mouse colon was not sufficient to induce these human IL-18 levels. The reasons why NLRC4^V341A/V341A^ mice produce less IL-18 than AIFEC patients thus remains unknown but are likely multifactorial. In line with their only moderately elevated IL-18 production, NLRC4^V341A/V341A^ mice showed no signs of spontaneous inflammation and did not develop detectable intestinal pathology. This suggested that IL-18 levels in NLRC4^V341A/V341A^ mice were not sufficiently high, or alternatively that endogenous antagonists prevented IL-18-mediated pathogenic effects. In this respect, considering the effective treatment of a critically ill neonatal AIFEC patient with recombinant IL-18BP (5), it is quite remarkable that additional IL-18BP deletion did not allow IL-18 to provoke spontaneous colitis in NLRC4^V341A/V341A^ mice. This shows that the absence of clinical signs in NLRC4^V341A/V341A^ mice does not result from the inability of their IL-18 levels to surpass endogenous inhibition by IL-18BP. Instead, additional yet to be identified triggers may be needed to unleash the pathogenic actions of IL-18 in NLRC4^V341A/V341A^ mice. In line with this hypothesis, also previously described NLRC4^T337S^-expressing mice that displayed much higher IL-18 serum levels than NLRC4^V341A/V341A^ mice did not develop spontaneous autoinflammatory pathology (14). Moreover, since AIFEC patients show chronically elevated IL-18 levels also in between flares, elevated IL-18 levels appear to require additional cues to drive disease in AIFEC patients as well (4–7). Therefore, it is likely that the absence of pathology in NLRC4^V341A/V341A^ mice is not merely due to their low IL-18 levels but also involves insufficient induction of additional factors that could cooperate with IL-18 to induce inflammation. For instance, evaluating IL-18-mediated induction of IFNγ in NLRC4^V341A/V341A^ mice could be informative to better understand the reasons why these mice did not develop autoinflammation.

Therefore, we next evaluated the role of commensal as well as infectious gastrointestinal bacteria as potential triggers of IL-18-mediated pathogenic activities. Interestingly, neonatal-onset enterocolitis spontaneously normalizes after the first year of life in surviving AIFEC patients (3, 4). This led to the hypothesis that early gut colonization may promote enterocolitis development in AIFEC patients, while maturation of the gut microbiota could lead to spontaneous resolution. In accordance with a role for the gut microbiota composition regulating AIFEC severity, a recent study showed that a fecal microbiota transplant improved clinical symptoms in an NLRC4^I343N^-expressing NLRC4-AID patient (34). However, our observations in GF mice showed that NLRC4^V341A^ expression elevated both systemic and intestinal IL-18 levels independently of the microbiota. In addition, we observed increased levels of cleaved IL-18 in whole colon and ileum of GF NLRC4^V341A/V341A^ mice, while we could only observe increased levels of pro-IL-18 in colon IECs of NLRC4^V341A/V341A^ mice housed in SPF conditions. These paradoxical observations likely relate to the higher IL-18 expression in SPF mice compared to GF mice, consistent with reports showing that the microbiota induce IL-18 in the gastrointestinal tract (27–30), making it more difficult to detect NLRC4^V341A^-induced IL-18 cleavage in SPF conditions as compared to GF conditions. Thus, intestinal inflammasome activation leading to IL-18 maturation in NLRC4^V341A/V341A^ mice appears to be masked by the gut microbiota. Nevertheless, the observed intestinal IL-18 maturation in GF NLRC4^V341A/V341A^ mice as well as the more specific ELISA-based detection of mature IL-18 in serum of GF and SPF NLRC4^V341A/V341A^ mice suggests that the *in vivo* IL-18-inducing molecular mechanisms employed by both human and mouse NLRC4^V341A^ at least partially rely on NLRC4 inflammasome activity. However, besides inflammasome-mediated IL-18 maturation, we also observed transcriptional upregulation of pro-IL-18 in colon IECs of SPF NLRC4^V341A/V341A^ mice that could contribute to increased IL-18 production in these mice. Although transcriptional coactivator functions have been reported for other members of the NLR family such as CIITA and NLRC5 (35, 36), to our knowledge such roles have not been described for NLRC4. Since increased pro-IL-18 mRNA levels were observed specifically in colon IECs but not in ileum IECs nor in BMDMs of NLRC4^V341A/V341A^ mice it seems rather unlikely that the V341A mutation confers a general transcriptional activator function to the NLRC4 protein. Instead, a low-grade inflammatory environment caused by IL-18 in the colon of NLRC4^V341A/V341A^ mice might enhance pro-IL-18 expression from colon IECs. As IL-18 is known to induce NF-κB activation via MyD88 signaling (37), this could be created by autocrine IL-18 stimulation of colon IECs. Alternatively, indirect effects of IL-18 via the production of downstream inflammatory factors such as IFNγ could upregulate pro-IL-18 expression in colon IECs (38). However, future research will be needed to clarify how NLRC4^V341A^ expression provokes transcriptional upregulation of pro-IL-18 and whether this may contribute to its effects in AIFEC patients.

While our observations in GF mice unequivocally showed that microbiota are not required to provoke production of mature IL-18 in NLRC4^V341A/V341A^ mice, the lack of colitis in either GF or SPF NLRC4^V341A/V341A^ mice suggested that additional bacterial triggers could be needed to allow IL-18 pathogenic activities. We therefore infected NLRC4^V341A/V341A^ mice orally with *S.* Typhimurium. Although NLRC4^V341A/V341A^ mice did not show altered susceptibility to this typhoid fever-like disease (23, 24), it should be noted that the NLRC4 inflammasome is not solely responsible for responding to this systemic infection. Indeed, *S.* Typhimurium dissemination was not altered in Nlrc4^-/-^ mice but was increased in Nlrc4^-/-^Nlrp3^-/-^ mice (39), raising the possibility that NLRC4^V341A^-induced inflammasome activation does not sufficiently augment Nlrp3 responses to alter the susceptibility of NLRC4^V341A/V341A^ mice to *S.* Typhimurium. It would therefore be interesting to assess the effect of NLRC4^V341A^ expression in *S.* Typhimurium-infected Nlrp3^-/-^ mice in future research. To more specifically evaluate NLRC4^V341A^ effects in a gastrointestinal infection model leading to IL-18 production from IECs, we infected NLRC4^V341A/V341A^ mice with *C. rodentium*, as NLRC4 responses in non-hematopoietic cells were shown to restrict colitis induced by this enteropathogen (26). In addition, canonical inflammasome-induced IL-18 production was shown to correlate with survival of mice susceptible to a lethal *C. rodentium* infection (13). However, *C. rodentium*-infected NLRC4^V341A/V341A^ mice did not display altered intestinal IL-18 levels nor altered host defense in comparison with *C. rodentium*-infected NLRC4^WT/WT^ mice. Thus, NLRC4^V341A^ expression does not alter the *in vivo* host defense against the NLRC4-activating *C. rodentium* and *S.* Typhimurium pathogens. However, it remains possible that other infectious triggers could be responsible for inducing disease in NLRC4^V341A/V341A^ mice. In this respect, both IL-18^tg^ and IL-18BP^-/-^ mice that harbor increased IL-18 activities develop more severe MAS upon repeated TLR9 stimulation (12, 14), suggesting that perhaps CpG DNA could represent a relevant PAMP for triggering disease in NLRC4^V341A/V341A^ mice. Future research in NLRC4^V341A/V341A^IL-18BP^-/-^ mice could investigate this possibility.

Overall, our observations in NLRC4^V341A/V341A^ mice are very reminiscent to a prior report on mice expressing the AIFEC-associated NLRC4^T337S^ variant (14). Like NLRC4^V341A/V341A^ mice, these NLRC4^T337S^ mice showed elevated IL-18 levels without detectable inflammatory pathologies and were not more susceptible to various challenges such as endotoxemia, Dextran Sodium Sulfate colitis, and infection with abortive or chronic Lymphocytic Choriomeningitis Virus (14). Interestingly, this study convincingly showed that the increased systemic IL-18 levels in NLRC4^T337S^ mice derived from the intestinal epithelium by specifically deleting IL-18 in IECs (14). Consistent herewith, we observed that colonic IECs from NLRC4^V341A/V341A^ mice under SPF conditions showed increased pro-IL-18 production, while GF NLRC4^V341A/V341A^ mice showed increased mature IL-18 levels in both colon and ileum. This suggests that the increased serum IL-18 levels seen in NLRC4^V341A/V341A^ mice might also originate from IECs. Taken together, these two AIFEC mouse models suggest that IECs may be the cellular IL-18 source in AIFEC patients (4–7). However, it cannot completely be ruled out that other cells may also contribute to the elevated IL-18 levels in these patients. Indeed, peripheral blood derived macrophages from AIFEC patients showed enhanced spontaneous IL-18 secretion, possibly adding to the increased IL-18 levels in the circulation (4, 6). However, this macrophage inflammasome hyperactivity was not recapitulated in neither NLRC4^V341A/V341A^ nor NLRC4^T337S^ BMDMs (14). Interestingly, in contrast to these AIFEC-associated NLRC4 variants, murine macrophages expressing the FCAS-associated NLRC4^H443P^ variant did display enhanced inflammasome activation as observed in human macrophages (40). Although further research is needed, these observations suggest that murine and human NLRC4 variants may employ very specific differences in the molecular signaling mechanisms leading to inflammasome activation.

Taken together, in this study we characterized a novel AIFEC mouse model expressing the NLRC4^V341A^ variant, thereby supporting observations in previously described NLRC4^T337S^ mice that AIFEC-associated NLRC4 variants elevate intestinal and systemic IL-18 levels. In addition, we show that NLRC4^V341A^ expression induces IL-18 production independently of the microbiota, and that this variant does not induce pathologies even in the absence of IL-18BP. This novel NLRC4^V341A^ mouse model sets the stage for further investigation of the triggers and pathogenic mechanisms involved during AIFEC development in humans.

## Data availability statement

The original contributions presented in the study are included in the article/supplementary material. Further inquiries can be directed to the corresponding author.

## Ethics statement

The animal study was approved by Ethical Committee Animal Experimentation VIB site Ghent - Ghent University - Faculty of Sciences. The study was conducted in accordance with the local legislation and institutional requirements.

## Author contributions

EE: Conceptualization, Formal Analysis, Writing – original draft, Writing – review & editing, Investigation, Methodology. TA: Formal Analysis, Investigation, Methodology, Writing – review & editing. HG: Writing – review & editing, Investigation, Methodology, Resources. DD: Investigation, Methodology, Writing – review & editing, Resources. CG-G: Resources, Writing – review & editing, Methodology. VA: Resources, Writing – review & editing. LV: Resources, Writing – review & editing. CG: Resources, Writing – review & editing. ML: Resources, Writing – review & editing. GvL: Supervision, Writing – review & editing. AW: Conceptualization, Formal Analysis, Funding acquisition, Project administration, Supervision, Writing – original draft, Writing – review & editing.
